# The Story of the Insane from Year to Year

**Published:** 1904-12-31

**Authors:** 


### THE STORY OF THE INSANE FROM YEAR TO YEAR.

### Seventeenth Annual Series.

### SURREY COUNTY ASYLUM AT BROOKWOOD.

On January 1st this asylum contained 1,070 patients, and on January 31st the number had risen to 1,265. There were 296 first admissions, 37 readmissions, and 120 retransfers, 95 recoveries, 23 discharged relieved, and 111 died. The total number under treatment was 1,523, and the average daily number resident was 1,180. The recoveries were 30·52 per cent. of the admissions, and the deaths were 9·40 per cent. of the average number resident. Cerebral and spinal diseases account for 53 of the deaths, consumption for 14, other lung diseases for 7 and colitis for 1. Of the year's admissions mental anxiety is supposed to have been the cause of the insanity in 41, religion in 11, domestic trouble in 23, intemperance in drink in 60, hereditary influence in 60, old age in 34. In 40 the insanity was congenital, and in 86 no cause was known. The additions to the asylum were opened during the summer and those patients who had been boarded out at the asylums of Bristol, Gloucester, Three Counties, and Sussex, were retransferred to Brookwood. The asylum was quite large enough before these new wards were built, and to add to an asylum which already contained over 1,000 beds can only be looked upon as a blunder, and probably an expensive blunder. A reservoir capable of containing 750,000 gallons of water has been made, and also a new coal-store which will hold 425 tons. Both these are most useful if not necessary adjuncts. Attendant Hobson received a pension of £60 a year, attendant Wyndebank one of £45, William Trower, the fitter, one of £65, and gardener-attendant Daborne one of 14s. a week. "The committee desire to express their approval of the management of the asylum by the medical superintendent."

